# Exploration of the thoracoscopic treatment of esophageal atresia under high-frequency ventilation

**DOI:** 10.3389/fped.2022.1066492

**Published:** 2022-12-22

**Authors:** Chao Zheng, Yu Lin, Yuanbin He, Yong Shen, Jiansen Fan, Yifan Fang

**Affiliations:** ^1^ Department of Pediatric Surgery, Fujian Children's Hospital (Fujian Branch of Shanghai Children's Medical Center), Fuzhou, China; ^2^ College of Clinical Medicine for Obstetrics & Gynecology and Pediatrics, Fujian Medical University, Fuzhou, China

**Keywords:** thoracoscopy, esophageal atresia, high-frequency oscillatory ventilation, HFOV, endoscopic

## Abstract

**Objective:**

Explore the feasibility and safety of thoracoscopy in the treatment of esophageal atresia under high-frequency oscillatory ventilation (HFOV) mode.

**Methods:**

This was a single-center retrospective analysis. A total of 24 children were divided into the HFOV and the No-HFOV group. The demographic information, surgical results and relevant experience were analyzed.

**Results:**

All patients in the HFOV group underwent thoracoscopic esophageal atreplasty with a mean operation duration of 165.8 ± 33.9 min. Two patients had postoperative anastomotic leakage, which was cured after conservative treatment. One child had a recurrent tracheoesophageal fistula, which was closed after endoscopic cauterization. The mean postoperative mechanical ventilation time was 8.83 ± 8.02 days. There was no return of anastomotic leakage or r-TEF after oral feeding. Furthermore, there was no significant difference between the NO-HFOV and the HFOV groups except for the operation time where the HFOV group was shorter than that of the NO-HFOV group.

**Conclusion:**

Thoracoscopic esophageal atresia anastomosis under HFOV ventilation is feasible for patients with severe pulmonary infection, heart malformation, such as patent ductus arteriosus, ventricular septal defect, and poor anesthesia tolerance, but the long-term prognosis still needs further study in a large sample size.

## Introduction

Esophageal atresia (EA) is a malformation caused by a disruption of the vacuolar phase of esophageal development during the embryonic stage, and frequently leads to a tracheoesophageal fistula (TEF) because the esophagus and trachea are not completely separated (1, 2). The incidence of EA is approximately 2.4/10,000 (3, 4). Moreover, it is a malformation that needs immediate attention during the newborn stage. The degree of treatment is reflected in the success rate of esophageal atresia anastomosis and the postoperative quality of life. Pneumonia and other congenital abnormalities are common in children with esophageal atresia. To improve the prognosis of children, intraoperative stabilization is crucial. Infants who have respiratory failure and aspiration pneumonia are now frequently given respiratory assistance using high-frequency oscillatory ventilation (HFOV) (5, 6). However, its usage in intraoperative respiratory support is infrequently described, and it is more implicated in perioperative support for congenital diaphragmatic hernia or congenital heart disease (7–9). Due to the mechanism of high-frequency oscillatory ventilation, there are still obstacles in performing thoracoscopic procedures in HFOV, and relatively few published studies exist on this topic. In this study, a retrospective examination of children with esophageal atresia who were treated with high-frequency oscillatory ventilation-assisted thoracoscopy at Fujian Children's Hospital from Oct 2019 to Oct 2022 was undertaken to determine the technique's applicability.

## Patients and method

### Patients

HFOV group: Patients receiving high-frequency oscillating ventilation-assisted thoracoscopic therapy from October 2019 to October 2022 were included. Newborns with an isolated TEF and without EA were excluded. The cases that were converted to open repair were not reviewed.

No-HFOV group: Patients who underwent thoracoscopic esophageal atresia in our hospital from October 2019 to October 2022 under the non-HFOV ventilation mode were randomly selected as the control group. The cases that were converted to open repair were not reviewed. The demographic information and surgical status of children in both groups were collected.

### Statistical analysis

The chi-square test was used for categorical data, and the Mann‒Whitney U test was used for nonparametric data. Data are expressed as the mean (range); *P* < 0.05 was considered significant.

The Ethics Committee at Fujian Children's Hospital approved this study, and all of the children's guardians provided informed consent. This study conforms to the Helsinki Declaration of 1975 (Revised in 1983).

### Surgical method

For surgery, the patient was prone with the right side raised 40°. A 5 mm trocar was inserted into the 5th intercostal space of the right subscapular angle. Under thoracoscopic guidance, two 3 mm trocars were inserted into the right midaxillary line's 4th and 6th intercostal spaces. The parietal pleura on the posterior mediastinal esophageal surface was split and exposed the posterior mediastinum and azygos vein. We continued to free the distal esophagus to the fistula. Two Hem-Lock clamps were used to close the fistula. The distal and proximal sides of the esophagus were unrestricted, which allowed both ends to be brought together without tension. The fistula was severed diagonally at 45 degrees and approximately 1 cm away. The proximal blind end of esophageal atresia was clipped.

The 5-0 absorbable thread was utilized to anastomose the distal and proximal ends of the esophagus.

### Anesthesia method

The children in the high-frequency ventilation group were all ventilated in HFOV mode; the frequency was 7–10 Hz, the amplitude was 15–20 mmHg, and the mean airway pressure (MAP) was 15 cm H20. The pH, Pao2, and PaCO2 values at different time points (as shown in Table 1) were compared, and ventilator parameters were adjusted based on the results combined with the transcutaneous CO2 readings.

**Table 1:** HFOV group blood gases.

	Mean	Range
Ph	7.25 ± 0.1	7.08–7.45
pCO2 (mmHg)	54.7 ± 9.9	44–82.8
pO2 (mmHg)	95.1 ± 40.9	29–237

## Results

This study included a total of 24 children as follows: 12 in the HFOV group (10 males and 2 females) and 12 in the No-HFOV group (7 males and 5 females). To confirm the diagnosis of esophageal atresia, all patients underwent an esophagography and chest CT. Additionally, each patient underwent thoracoscopic esophageal anastomosis.

In the HFOV group, the birth weight ranged from 1,550 to 3,700 g with a mean of 2795.4 ± 696.9 g. The children included 1 case of type IIIa and 11 cases of type IIIb esophageal atresia. Eight patients underwent mechanical ventilation with tracheal intubation, and four patients had noninvasive ventilation prior to the surgery. Every child had at least one abnormality, including cardiovascular malformation, urinary system malformation, and anorectal malformation (as shown in Table 2). The average surgery time was 165.8 ± 33.9 min, and the average intraoperative blood loss was 8.92 ± 10.93 ml. Intermittent intraoperative blood gas samples and percutaneous CO2 measurements confirmed adequate ventilation and oxygenation throughout the operation (as shown in Table 1). After surgery, the length of tracheal intubation in this group was 8.83 ± 8.02 days. Nasal feedings were initiated 11.5 ± 13.7 days after surgery, while oral feeding was initiated 15.7 ± 14.1 days following surgery. The weight at discharge was 3,142.08 ± 380.8 g.

**Table 2:** Patient demographics, preoperative status.

No	Gender	Admission Age (h)	Birth weight (g)	Tire number	Prenatal diagnosis	Combined disease
1	Male	41	1,550	G4P1	Yes	Hypospadias Penoscrotal transposition
2	Male	20	1,655	G4P4	Yes	Respiratory failure, PDA congenital solitary kidney
3	Female	18	2,700	G1P1	No	PDA, Polycystic kidney Anal atresia
4	Female	10	3,010	G3P2	No	PFO
5	Male	37	3,650	G1P1	No	Neonatal dystonia, PDA Respiratory muscle paralysis
6	Male	28	3,620	G2P1	No	Neonatal dystonia, PFO
7	Male	42	3,700	G5P3	No	Neonatal pneumonia Asymmetric crying syndrome
8	Male	7	2,400	G5P1	No	Neonatal pneumonia Right thumb polydactyly
9	Male	11	2,800	G3P2	No	PDA Scalp hematoma
10	Male	24	3,000	G2P1	No	PDA Respiratory failure
11	Male	72	2,700	G2P2	No	PFO Meconium aspiration syndrome
12	Male	4	2,760	G3P2	No	Neonatal pneumonia PDA

None of the children in the No-HFOV group received mechanical ventilation before surgery. The birth weight of the patients in this group was 2,530.3 ± 450.2 g. There were 2 occurrences of esophageal atresia type IIIa and 10 cases of type IIIb. The procedure lasted 222.3 ± 80.3 min, intraoperative blood loss was 6.33 ± 4.7 ml, postoperative intubation lasted 9.5 ± 11.5 d, nasogastric feeding commenced at 10.3 ± 6.5 d postoperatively, and oral feeding was administered 21.9 ± 14.5 d postoperatively.

Two patients in the HFOV group suffered anastomotic leakage, which was healed with conservative treatment; one patient developed r-TEF, which was treated with endoscopic cautery (as shown in Table 3). Two children in the NO-HFOV group suffered anastomotic leakage, which was successfully treated with conservative measures. Patients in both groups were released without incident, and there was no return of anastomotic leakage or r-TEF after oral feeding. The operation time of the children in the No-HFOV group was statistically longer than that of the children in the HFOV group. In addition, there were no significant differences between the two groups in terms of blood loss, postoperative extubation, or oral feeding time (as shown in Table 4).

**Table 3:** Surgical information.

No	Operation Time (min)	Type	Operative bleeding (ml)	Distance (cm)	Postoperative mechanical ventilation time (d)	Postoperative complications
1	160	IIIb	5	1.5	14	r-TEF
2	150	IIIb	5	1.3	7	-
3	190	IIIa	5	5	5	-
4	137	IIIb	3	1.6	8	-
5	115	IIIb	2	1	20	Anastomotic leakage
6	133	IIIb	40	1.5	2	-
7	200	IIIb	20	1	1	-
8	195	IIIb	10	1	16	-
9	164	IIIb	5	1.5	2	-
10	200	IIIb	3	2	4	-
11	218	IIIb	3	1.5	25	Anastomotic leakage
12	128	IIIb	6	1	2	-

**Table 4:** Comparison with the control group.

	NO-HFOV	HFOV	*P*
Number of patients	12	12	NA
Sex (male/female)	7/5	10/2	NA
Prenatal diagnosis (yes/no)	4/8	2/10	*P* > 0.05
Birth weight (g)	2,530.3 ± 450.2	2,795.4 ± 696.9	*P* > 0.05
Surgery time (min)	222.3 ± 80.3	165.8 ± 33.9	*P* = 0.045
Operation bleed (ml)	6.33 ± 4.7	8.92 ± 10.93	*P* > 0.05
Postoperative intubation time (d)	9.5 ± 11.5	8.83 ± 8.02	*P* > 0.05
Postoperative nasal feeding time (d)	10.3 ± 6.5	11.5 ± 13.7	*P* > 0.05
Oral feeding time (d)	21.9 ± 14.5	15.7 ± 14.1	*P* > 0.05
Discharged weight (g)	3,252.5 ± 887.2	3,142.08 ± 380.8	*P* > 0.05

## Discussion

EA frequently causes an aspiration and reflux pneumonia in children. Postural therapy, sputum suction, and if necessary, mechanical breathing support are the primary therapeutic techniques (10, 11). When normal frequency ventilation fails to establish hemodynamic stability, high-frequency ventilation for respiratory support is frequently required (12). High-frequency ventilation employs high respiratory rates (generally greater than 150 breaths per minute) to provide tidal volumes that are typically less than the anatomical dead space. It has been used effectively to treat severe respiratory distress syndrome, bronchopleural fistula, pulmonary interstitial emphysema, and other air leakage-related complications (13–15). The next question is whether the operation is performed under HFOV, or should there be a delay and a switch to a mode of constant frequency ventilation for stabilization, followed by the performance of the surgery? There are no explicit guidelines or consensuses that provide guidance. Notably, delaying esophageal atresia anastomosis may result in an uncontrolled reflux pneumonia and increase the difficulty of subsequent anastomosis; therefore, our recommendation is to perform esophageal atresia anastomosis under HFOV as soon as the patient can achieve stable hemodynamics with the support of HFOV.

Additionally, it should be emphasized that more than 50% of patients with EA have other congenital malformations, some with two or more anomalies (such as VACTERL syndrome), and even some with neuromuscular diseases and complex congenital cardiac anomalies (16, 17). In this study, a patient with a neuromuscular disorder caused by a genetic mutation exhibited respiratory muscle weakness. HFOV ventilation was useful in maintaining respiratory function and hemodynamic stability during the operation. This breathing approach is also appropriate for children with congenital cardiac disease, such as those with a large diameter PDA.

**Figure 1:**
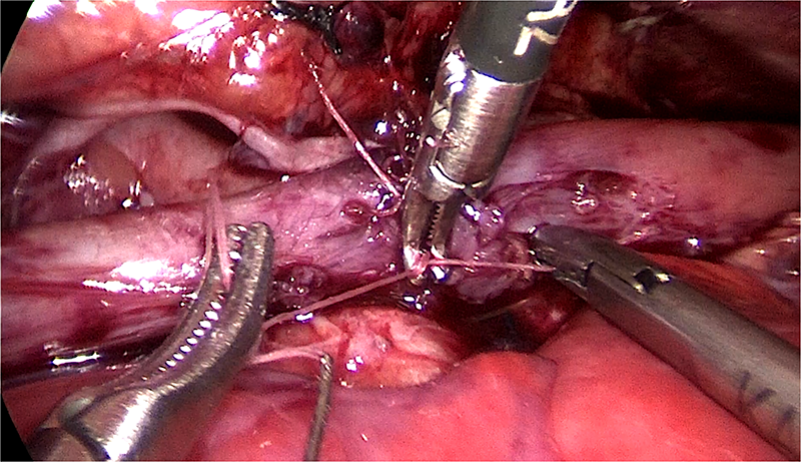
An auxiliary clamp can be positioned at the 5th intercostal gap of the subscapular angle.

**Figure 2:**
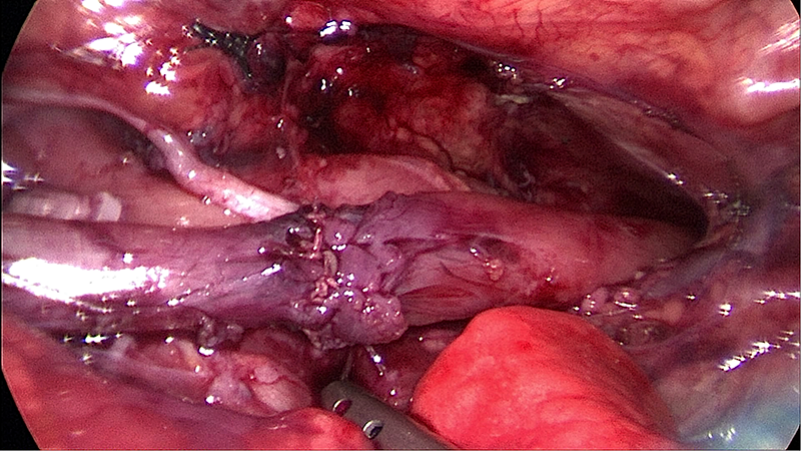
Esophageal atresia anastomosis.

The rapid vibration of the operative field from this ventilation is the primary distinction between HFOV support and conventional ventilation. Requirements for thoracoscopic surgical expertise have significantly expanded. The typical procedure of HFOV group lasted 165.8 ± 33.9 min on average, which was a considerable duration and indicated the difficulties that the surgeon faced when performing the operation with this ventilation technique. Thus, our perspective is that if you are inexperienced, the use of electrocautery and electrocoagulation can be decreased, and the separation method based mostly on blunt separation can be increased and result in less collateral damage. After ligation of the fistula, the vibration frequency can be decreased and reduce the difficulty of anastomosing the distal and proximal esophagus. For type I and type IIIa esophageal atresia, an auxiliary clamp or fascial closure device can be positioned at the 5th intercostal gap of the subscapular angle to draw the suture and to aid the anastomosis (as shown in Figures 1,2).

Due to the use of active expiration with a high-frequency oscillator, the mean airway pressure was maintained at a high level during the whole respiratory cycle and prevented airway collapse during expiration (18, 19). Because of the airway pressure, the fistula and peri-esophageal tracheal tissue were more precisely separated during treatment. Given that the increase in intrathoracic carbon dioxide pressure and airway pressure impacts the child's hemodynamic stability and that the child's fundamental condition is not stable, it is essential to know when to stop the procedure. If the blood oxygen saturation falls below 80% or the end-tidal carbon dioxide partial pressure increases above 55 mmHg (1 mmHg = 0.113 kPa), the surgery is stopped and the carbon dioxide intake is terminated in cooperation with the anesthesiologis. When the situation improves, the operation will continue.

However, it is vital to emphasize that this sort of ventilation demands the anesthesiologist's cooperation. Adjustment of this HFOV breathing technique must be personalized. Before ligating the fistula, oscillation frequency and airway pressure must be maintained at a high range. After the fistula has been closed, bilateral lung ventilation can be enabled, in contrast with one-lung ventilation that occurs during conventional ventilation. Then, the oscillation frequency can be reduced to facilitate anastomosis. With this method of ventilation, it is also difficult to deliver volatile medications; typically, total intravenous anesthesia (TIVA) procedures are required and coupled with muscle relaxants and high-dose anesthetic infusions. Moreover, cardiac output and venous return may diminish during breathing due to the increased average airway pressure. Intraoperative hemodynamic measurements depend on arterial blood gas or percutaneous (TC) measurements of carbon dioxide. Therefore, TC-CO detection during NICU monitoring and the correlation of TC-CO2 with baseline arterial blood gasfor each patient.

In comparing children in the NO-HFOV group with those in the HFOV group, there were no significant differences in postoperative complications or extubation time. However, the children in the No-HFOV group had longer operation time than those in the HFOV group. Most children in the HFOV group were in poor condition before surgery and needed mechanical ventilation support, which indicated that thoracoscopic esophageal atresia was safe and feasible under HFOV ventilation. However, the lower operating time in the HFOV group may be a result of the surgeons' enhanced experience. In addition, future large-scale testing is still required due to the small sample size of the study's patients and the likelihood of individual variances influencing the findings.

Some studies have found that although the application of mechanical ventilation can improve the treatment effect of children with respiratory failure, mechanical ventilation treatment can also affect the prognosis of children and their quality of life and can even lead to the occurrence of death (20, 21). All children included in this study were discharged successfully without significant dyspnea and choking after discharge, but their long-term recovery of respiratory function and quality of life still needs further follow-up.

## Conclusions

In conclusion, thoracoscopic esophageal atresia anastomosis under HFOV ventilation is feasible for children with EA complicated with severe pulmonary infection, cardiac malformation, such as patent ductus arteriosus, ventricular septal defect, and poor tolerance to anesthesia and can maintain their intraoperative respiratory function and hemodynamic stability. However, the requirements for surgeons and anesthesiologists are great and demand multidisciplinary cooperation. Additional large sample studies are necessary to determine the long-term prognosis.

## Data Availability

The original contributions presented in the study are included in the article/**Supplementary Material**, further inquiries can be directed to the corresponding author/s.
